# A Novel Botanical Combination Attenuates Light-Induced Retinal Damage through Antioxidant and Prosurvival Mechanisms

**DOI:** 10.1155/2020/7676818

**Published:** 2020-03-13

**Authors:** Juntao Kan, Junrui Cheng, Jun Guo, Liang Chen, Xue Zhang, Jun Du

**Affiliations:** ^1^Nutrilite Health Institute, 720 Cailun Road, Shanghai 201203, China; ^2^Department of Plants for Human Health Institute, North Carolina State University, 2501 Founders Drive, NC 27695-7601, USA

## Abstract

The prevalence of light-induced eye fatigue is increasing globally. Efficient regimen for mitigating light-induced retinal damage is becoming a compelling need for modern society. We investigated the effects of a novel combination of lutein ester, zeaxanthin, chrysanthemum, goji berry, and black currant extracts against retinal damage. In the current work, both *in vitro* and *in vivo* light-induced retinal damage models were employed. Animal study showed that under strong light exposure (15000 lx for 2 hours), the *a*-wave and *b*-wave from electroretinogram were significantly decreased. Treatment with the combination significantly restored the decrease for *b*-wave under high- and low-stimulus intensity. Histological analysis reported a substantial decrease in the outer nuclear layer (ONL) thickness in the model group, while the supplementation with the combination significantly improved the ONL thickness. To further explore the underlying mechanism of the protective effects, we utilized ARPE-19 retinal pigment epithelial cell line and found that strong light stimulation (2900 lx for 30 minutes) significantly increased phosphorylation of p38 and JNK and decreased HIF expression. Intriguingly, chrysanthemum, black currant extracts, lutein ester, and zeaxanthin significantly decreased the phosphorylation of p38 and JNK, while chrysanthemum, goji berry, black currant extracts, and lutein ester restored HIF expression. The botanical combination can alleviate light-induced retina damage, potentially through antioxidant and prosurvival mechanisms.

## 1. Introduction

Eye fatigue, also defined as asthenopia, is a common condition in both adults and children and can result from a variety of causes, including uncorrected refractive errors, imbalance of extraocular muscles, accommodative impairment, and improper lighting [1]. It is frequently associated with situations where the accommodative and vergence processes are more intense, exemplified by long-period looking at video display units (VDUs), e.g., computer, mobile phone, and iPad [1]. Blue light emitted from VDUs can damage retinal cells, which has been reported to be related to eye fatigue [2–4]. With a global increasing rate of asthenopia paralleled with more frequently used VDUs at different stages of life [1, 5–7], it is important to develop an effective method to mitigate asthenopia-related manifestations and curb the progressing of retinal damage.

Retinal pigment epithelium (RPE) constitutes a single layer of tissue in the vertebrate eye between Bruch's membrane and the photoreceptor layer of the neural retina [8]. RPE cells are packed with mitochondria and engage actively in oxidative metabolism to support photoreceptor function; in addition, they are responsible for the elimination of metabolic end products from the photoreceptors [9, 10]. Conditions such as oxidative stress, ceruloplasmin and hephaestin deficiency, and extra iron accumulation can cause RPE apoptosis and retinal degeneration in humans [11–13]. Therefore, it has been widely recognized that preventing RPE damage is critical in maintaining ocular health [12, 14, 15].

It has been hypothesized that nutrition could play a vital role in overall ocular health [16, 17]. Various nutrients are needed for maintaining eye functions and may therefore be involved in preventing eyes from harmful light exposures [17–20]. Lutein and zeaxanthin are xanthophyll carotenoids that are concentrated in the macula and are known for their roles in antioxidation [19–21]. One systemic review with meta-analysis of twenty randomized controlled trials (RCTs) revealed a proportional increase of macular pigment optical density with lutein and zeaxanthin supplementation [22], indicating beneficial efficacy of supplemented lutein and zeaxanthin intake in improving ocular health. Accumulating evidence has shown that black currant (*Ribes nigrum L.*) is beneficial for visual functions potentially by increasing ocular blood flows to mitigate eye fatigue symptoms [23, 24]. One RCT by Yagi et al. reported that daily oral administration of lutein (5 mg), zeaxanthin (1 mg), and black currant extract (200 mg) for 2 weeks significantly alleviated participants' eye fatigue senses in a strenuous visual task and increased eye fixation-related brain potential amplitude as an indicator of enhanced visual attention [25]. Chrysanthemum (*Chrysanthemum morifolium*) and goji berry (*Lycium barbarum L.*) are core herbs in traditional Chinese medicine remedies that classically used for protecting and nourishing liver and eye due to their antioxidant, anti-inflammatory, antiexcitotoxic, and antiapoptotic properties [26, 27]. The combination of these two ingredients, in particular, has been implicated in treating various vision-related diseases including glaucoma, ischemia/reperfusion, and age-related macular degeneration [26, 27].

In the current study, we combined lutein ester, zeaxanthin, extracts of black currant chrysanthemum, and goji berry into a mixed formula and aimed to investigate the curative efficacy of this novel combination against light-induced retinal damage in a rat model and its underlying molecular mechanism *in vitro*.

## 2. Materials and Methods

### 2.1. Materials and Reagents

Water extract of black currant with an extraction ratio of 4 : 1 was obtained from Diana Food (Antrain, France). Water extracts of chrysanthemum with an extraction ratio of 3 : 1 and goji berry with an extraction ratio of 3 : 1 were obtained from Novanat (Shanghai, China). Lutein ester powder with a spec of 10% lutein ester (bioactive equivalent to 5% free lutein) was obtained from BASF (Shanghai, China). Zeaxanthin powder with a spec of 5% zeaxanthin was obtained from DSM (Shanghai, China). The combination was prepared by Nutrilite Health Institute through mixture of 100 mg of black currant extract, 75 mg of chrysanthemum extract, 75 mg of goji berry, 120 mg of lutein ester powder, and 24 mg of zeaxanthin powder (daily intake for human). The vehicle was DMSO for *in vitro* study and soybean oil for animal study.

### 2.2. Cell Culture

ARPE-19, a human retinal pigment epithelial cell line, was purchased from American Type Culture Collection (Manassas, VA), and cultured in Dulbecco's modified Eagle's/Ham's F12 media (Invitrogen, Carlsbad, CA) supplemented with 10% fetal bovine serum (Invitrogen), 1% penicillin (100 U/mL), and streptomycin (100 mg/mL) (Invitrogen) at 37°C under a humidified 5% CO_2_ atmosphere.

### 2.3. Light Exposure In Vitro

**Figure 1:**
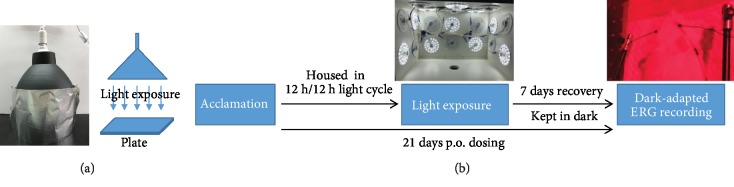
Flow charts of *in vitro* (a) and *in vivo* (b) studies.

Light-induced RPE cell damage model was established following the previously published paper with minor modification [15]. The integrated light-emitting diode system was purchased from Lvjing Photoelectricity Limited Company (Shenzhen, China) with a light spectrum of 430− 660 nm (Figure 1(a)). Light intensity was measured by TES-1330A illuminometer (TES Electrical Electronic Corporation, Taipei, Taiwan). The ARPE-19 cells were pretreated with 100 *μ*g/mL of different ingredients for 18 hours before exposed to the light (2900 lx) for 30 minutes. For control group, aluminum foil was placed on the plate to cover light exposure.

### 2.4. Animals

Male Sprague Dawley rats, 6-8 weeks, were purchased from SLAC (Shanghai, China). The animals were housed in a temperature and humidity-controlled room (22–23°C and 46–63%, respectively) and had free access to food and water. The experimental protocols were approved by the Institutional Animal Care and Use Committee of ChemPartner (Shanghai, China).

### 2.5. Light-Induced Retinal Damage Model

After one-week acclimation, the animals were randomly divided into four groups (8 rats for each group): (1) naïve control; (2) model+vehicle; (3) model+lutein (10 mg/kg); and (4) model+formula (65.7 mg/kg). The rats in groups 1-2 were given vehicle and those in groups 3-4 were given test articles by oral gavage daily for 21 days. Light-induced retinal damage model was established following the previously published paper with minor modification (Figure 1(b)) [15, 28]. The rats in groups 2-4 were dark adapted (60-100 lx) for 24 hours, and the pupils were dilated with 0.5% of tropicamide eye drops 20 minutes before light exposure. The rats were then exposed to diffuse, cool, white fluorescent light of 15000 lx for 2 hours, in which the light intensity and period were optimized by pilot study, in a cage with white walls (50 × 40 × 30 cm). After light exposure, the rats were kept in the dark for 7 days until further analysis.

### 2.6. Electroretinogram (ERG)

ERGs were recorded using a visual electrophysiology system ASP-2000AER (Kanghua Ruiming Science & Technology Co., Chongqing, China) to measure retinal function. The standard dark-adapted ERG signal was recorded at 7 days after light exposure. The rats were anesthetized with pentobarbital sodium (30 mg/kg) before the pupils were dilated with a topical application of 0.5% tropicamide and 0.5% amethocaine. After dark adaptation for more than 30 minutes, LED built-in contact lens electrode, reference electrode, and adjacent electrode were placed on the corneal surface, forehead with conductance ointment, and bitemporal, respectively. The luminous energy was calibrated using the internal calibration function of the LED stimulator. The responses were differentially amplified using a band-pass filter between 1 and 100 Hz for dark-adapted ERGs. Dark-adapted ERGs were recorded at a low stimulus intensity of 6.325*E* − 04 cd^∗^s/m^2^ and a high stimulus intensity of 6.325*E* − 03 cd^∗^s/m^2^.

### 2.7. Histological Analysis

The rats were sacrificed by euthanasia after ERGs recording, and then the eyes were enucleated and fixed with 4% paraformaldehyde before embedded into paraffin and cut into 4 *μ*m sections. Paraffin sections were stained with hematoxylin and eosin (H&E). For each section, digitized image of the entire retina tissue was scanned and analyzed using Aperio AT2 and ImageScope (Leica Biosystems Imaging Inc., Buffalo Grove, IL) at 20x magnification with 11951 × 13057 pixels. The outer nuclear layer (ONL) thickness was measured at 0.5, 1.0, 1.5, 2.0, 2.5, 3.0, 3.5, and 4.0 mm superior and inferior to the optic nerve head (ONH).

### 2.8. Western Blot

After light exposure, ARPE-19 cells were added with cell lysis buffer (Cell Signaling, Danvers, MA) to extract protein. Lysates were boiled with 5x loading buffer and then loaded onto sodium dodecyl sulfate-polyacrylamide gels (Bio-Rad, Hercules, CA). The separated proteins were transferred to polyvinylidene fluoride (PVDF) membranes (Millipore, Billerica, MA), then blocked with 5% nonfat dry milk in Tris-buffered saline with Tween-20. After incubated with primary antibodies of p-p38, p38, p-JNK, JNK, p-ERK, ERK, HIF, and GAPDH (Cell Signaling) at 4°C overnight, the PVDF membranes were then incubated with horseradish peroxidase- (HRP-) conjugated secondary antibodies at room temperature for 2 hours. The bands were developed by Immobilon™ Western Chemiluminescent HRP Substrate (Millipore) and imaged by ChemiDoc MP Imaging System (Bio-Rad).

### 2.9. Statistical Analysis

Statistical analysis was performed using SPSS 13.0 software (Chicago, IL). Quantitative data were presented as mean ± standard deviation (SD). Differences between groups were analyzed by one-way analysis of variance (ANOVA) with Dunnett's post hoc test. A significant effect was defined as *P* < 0.05.

## 3. Results

### 3.1. Protective Effects of the Combination on Retinal Function

**Figure 2:**
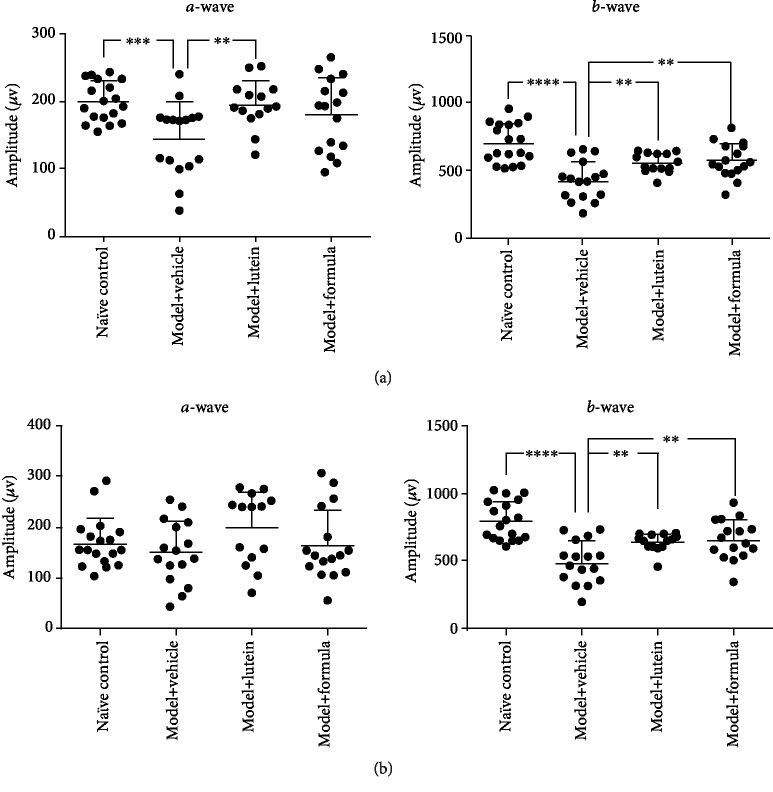
Electroretinogram (ERG). *a*-wave and *b*-wave of ERG under low-stimulus intensity of 6.325*E* − 04 cd^∗^s/m^2^ (a) and high-stimulus intensity of 6.325*E* − 03 cd^∗^s/m^2^ (b) were recorded to measure retinal function at 7 days after light exposure of 15000 lx for 2 hours. Rats were treated with vehicle (soybean oil), lutein (10 mg/kg), or formula (65.7 mg/kg) by oral gavage daily for 21 days before the recording. Data were presented as mean ± SD. *N* = 8. ^∗∗^*P* < 0.01, ^∗∗∗^*P* < 0.001, ^∗∗∗∗^*P* < 0.0001.

Compared with naïve control, strong light exposure of 15000 lx for 2 hours led to a significant decrease in the amplitude of ERG for *b*-wave (486.4 ± 158.4 *μ*V vs. 795.6 ± 144.5 *μ*V, *P* < 0.001) under high-stimulus intensity of 6.325*E* − 03 cd^∗^s/m^2^, and for both *a*-wave (143.9 ± 54.13 *μ*V vs. 199.3 ± 29.84 *μ*V, *P* < 0.001) and *b*-wave (423.4 ± 145.8 *μ*V vs. 704.2 ± 146.1 *μ*V, *P* < 0.001) under low-stimulus intensity of 6.325*E* − 04 cd^∗^s/m^2^ (Figure 2). Lutein at a dosage of 10 mg/kg, acted as positive control, effectively restored *b*-wave (637.2 ± 66.33 *μ*V vs. 486.4 ± 158.4 *μ*V, *P* < 0.01) under the high-stimulus intensity and for *a*-wave (194.8 ± 35.77 *μ*V vs. 143.9 ± 54.13 *μ*V, *P* < 0.01) and *b*-wave (556.0 ± 72.28 *μ*V vs. 423.4 ± 145.8 *μ*V, *P* < 0.01) under the low-stimulus intensity, suggesting the light-induced retinal damage model was well validated.

Treatment of the combination significantly restored the decrease for *b*-wave under the high-stimulus intensity (655.5 ± 149.6 *μ*V vs. 486.4 ± 158.4 *μ*V, *P* < 0.01) and under the low-stimulus intensity (571.5 ± 128.1 *μ*V vs. 423.4 ± 145.8 *μ*V, *P* < 0.01). *a*-wave was also moderately increased by the treatment, although not reaching statistical significance. There was no significant difference between lutein and formula groups. Such data suggested that our combination might be beneficial to retinal function.

### 3.2. Protective Effects of the Combination on Retinal Structure

**Figure 3:**
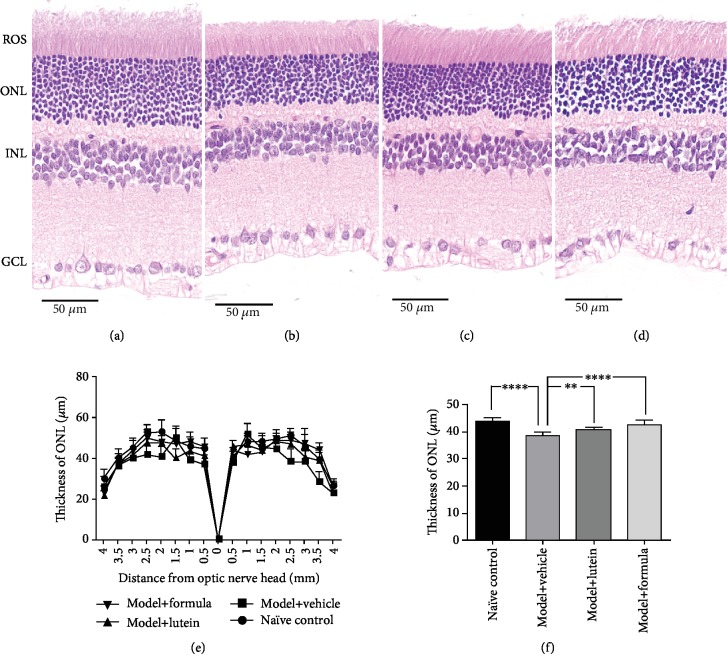
Histopathologic evaluation. After the ERG, the rats were sacrificed and the eye tissues were collected for retinal structure analysis. (a–d) Representative photos of H&E staining for naïve control, model+vehicle, model+lutein, and model+formula, respectively. (e) The thickness of outer nuclear layer (ONL) measured at 0.5, 1.0, 1.5, 2.0, 2.5, 3.0, 3.5, and 4.0 mm superior and inferior to the optic nerve head (ONH). (f) The mean of thickness value from (e). ROS: rod outer segment; INL: inner nuclear layer; GCL: ganglion cell layer. Data were presented as mean ± SD. *N* = 8. ^∗∗^*P* < 0.01, ^∗∗∗∗^*P* < 0.0001.

To investigate the efficacy of the combination on retinal structure, histological analysis was performed with H&E staining (Figures 3(a)–3(d)). Consistent with the ERG data, a pronounced decrease (38.56 ± 1.50 *μ*m vs. 44.18 ± 1.31 *μ*m, *P* < 0.001) in ONL thickness was seen in the model group (Figures 3(e)–3(f)). No other abnormal retinal morphology was observed. Treatment of both the combination (42.77 ± 1.67 *μ*m vs. 38.56 ± 1.50 *μ*m, *P* < 0.001) and lutein (40.75 ± 0.86 *μ*m vs. 38.56 ± 1.50 *μ*m, *P* < 0.01) improved the ONL thickness, suggesting protective effects of the combination on retinal structure.

### 3.3. Protective Mechanisms of the Ingredients in the Light-Induced Retinal Damage

**Figure 4:**
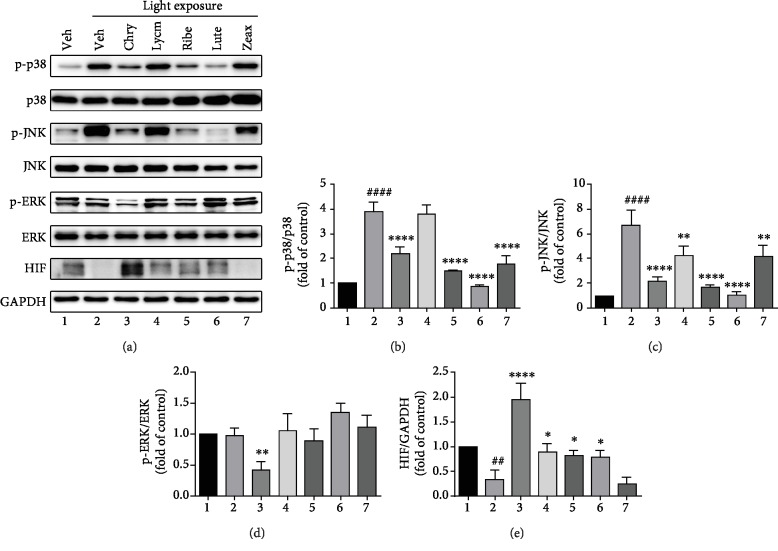
MAPK and HIF pathway. The ARPE-19 cells were pretreated with 100 *μ*g/mL of different ingredients for 18 hours before exposed to the light (2900 lx) for 30 minutes, and then protein was extracted from cell lysate and analyzed with Western blot. GAPDH was used as a loading control. (a) Representative blots. (b) Changes of p-p38/p38. (c) Changes of p-JNK/JNK. (d) Changes of p-ERK/ERK. (e) Changes of HIF. Data were presented as mean ± SD. ^∗^*P* < 0.05, ^∗∗^*P* < 0.01, ^∗∗∗∗^*P* < 0.0001 vs. light exposure with vehicle (lane 2). ^##^*P* < 0.01, ^####^*P* < 0.0001 vs. no light exposure with vehicle (lane 1). Experiments reaped at least three times.

Light exposure to the ARPE-19 cells led to increased (*P* < 0.001) phosphorylation of p38 and JNK as well as decreased (*P* < 0.01) HIF expression (Figure 4). Chrysanthemum significantly decreased (*P* < 0.01) the phosphorylation of p38, JNK, and ERK. In addition, black currant, lutein ester, and zeaxanthin also decreased (*P* < 0.01) the phosphorylation of p38 and JNK. Chrysanthemum, goji berry, black currant, and lutein ester significantly increased (*P* < 0.001 for chrysanthemum, *P* < 0.05 for others) HIF expression, and intriguingly, chrysanthemum elevated the expression to a much higher level than the control group.

## 4. Discussion

Individually, black currant, chrysanthemum, goji berry extracts, lutein ester, and zeaxanthin have been reported as active ingredient or nutrient against eye fatigue in multiple studies [20, 21, 23, 26, 27]. However, to date, no one has investigated the efficacy of the combination of these ingredients or nutrients on ocular health. We provided first evidence showing that a mix of the abovementioned regimen effectively protected against light-induced retinal damage. Nevertheless, we did not find superior efficacy of the combination over a single free lutein supplementation in both *in vivo* and *in vitro* studies, which is inconsistent with our hypothesis. This might be due to a lack of synergistic efficacy within our formula or because lutein ester is the key nutrient that provided the retinal protection in this model.

The ERG is a diagnostic test that detects the electrical responses generated by neural and nonneuronal cells in the retina when a light stimulus occurs and provides important diagnostic information on a variety of retinal disorders [29, 30]. Various ocular abnormalities may manifest themselves through decreased ERG *a*- and *b*-wave responses including eye toxicity, vitamin A deficiency, and asthenopia [29–31]. In the current work, we found a significant drop of both ERG *a*- and *b*-waves after exposing animals to light stimulus, which is a demonstration of successful animal modeling. Interestingly, by providing rats with the formula, their ERG *b*-wave restored from the post stimulus baseline, and the *a*-wave was also moderately increased, although not reaching statistical significance. Such result suggested that our formula improved the retinal function. Meanwhile, our histological analysis reported that the treatment of the formula increased the thickness of ONL, which contains cell bodies of rod and cone photoreceptors, showing that our formula was beneficial to retinal structure. These data were consistent with the ERG data.

Since RPE is closely connected to the photoreceptor layer of neural retina and supports photoreceptor function, we further investigated possible underlying mechanism of our formula in a retinal pigment epithelial cell line. Indeed, by performing the *in vitro* study, we found the treatment of individual constituent in our formula substantially decreased the phosphorylation of p38 and JNK proteins. JNK and p38 are members of MAP-kinases (MAPK) playing prominent roles in programmed cell death of the development of the vertebrate nervous system, of which the retina is a unique sensory component [32]. The reduced activation of p38 and JNK proteins in ARPE-19 cells indicated that the isolated regimen might be able to enhance retina cell survival by inhibiting the MAPK signaling. HIF is known as a transcription factor that facilitates cellular adaptation to hypoxia and ischemia and involves in antioxidative stress [33]. Recently, an increasing body of evidence has suggested that HIF may contribute to retinal neuroprotection by targeting a variety of genes such as vascular endothelial growth factor (VEGF) [34, 35] and Heme oxygenase 1 (HO-1) [36–38]. Our results showed that the individual constituent from the formula significantly increased the HIF expression in light-exposed ARPE-19 cells, indicating that the mixed ingredients might provide support to retinal structure and function by mitigating light-induced oxidative stress.

One limitation of our study was that we were not able to identify every single nutrient in the black currant, goji berry, and chrysanthemum extract we used in formulating our final supplement. However, since the previous studies that revealed protective efficacy of black currant, goji berry, and chrysanthemum on vision health employed whole food extract, rather than isolated constituent [25–27], we have good reason to believe that an entire extract that mimic whole food may provide superior protective efficacy compared to single nutrient, considering that interrelations between constituents in foods are significant [39].

## 5. Conclusions

In conclusion, our study provided first evidence that a mix of black currant, goji berry, chrysanthemum extract, lutein ester, and zeaxanthin was able to alleviate the retinal damage induced by light exposure. The underlying mechanism might be through a prosurvival mechanism by inhibiting MAPK signaling and an antioxidative mechanism by elevating HIF protein expression. Although both *in vitro* and *in vivo* studies of our current work support the beneficial efficacy of this novel formula in protecting against light-induced asthenopia, further human researches are warranted to validate its functionality in clinical practice.

## Data Availability

The data used to support the findings of this study are available from the corresponding author upon request.
